# Tea and coffee consumption and risk of acute stroke: The INTERSTROKE Study

**DOI:** 10.1177/17474930241264685

**Published:** 2024-07-31

**Authors:** Andrew Smyth, Graeme J Hankey, Peter Langhorne, Catriona Reddin, Danuta Ryglewicz, Annika Rosengren, Dennis Xavier, Michelle Canavan, Shahram Oveisgharan, Xingyu Wang, Patricio Lopez Jaramillo, Albertino Damasceno, Anna Czlonkowska, Helle Klingenberg Iversen, Fernando Lanas, Salim Yusuf, Martin O’Donnell

**Affiliations:** 1 Population Health Research Institute, McMaster University and Hamilton Health Sciences, Hamilton, ON, Canada; 2 HRB Clinical Research Facility Galway, University of Galway, Galway, Ireland; 3 Medical School, The University of Western Australia, Perth, WA, Australia; 4 Academic Section of Geriatric Medicine, Glasgow Royal Infirmary, University of Glasgow, Glasgow, UK; 5 Military Institute of Aviation Medicine, Warsaw, Poland; 6 Sahlgrenska University Hospital and Sahlgrenska Academy, University of Gothenburg, Gothenburg, Sweden; 7 St John’s Medical College and Research Institute, Bangalore, India; 8 RUSH Alzheimer Disease Research Center, RUSH University Medical Center, Chicago, IL, USA; 9 Beijing Hypertension League Institute, Beijing, China; 10 Masira Research Institute, Universidad de Santander, Bucaramanga, Colombia; 11 Facultad de Medicina Eugenio Espejo, Universidad UTI, Quito, Ecuador; 12 Eduardo Mondlane University, Maputo, Mozambique; 13 Institute of Psychiatry and Neurology, Warsaw, Poland; 14 Stroke Center, Rigshospitalet, University of Copenhagen, Copenhagen, Denmark; 15 Faculty of Medicine, Universidad de La Frontera, Temuco, Chile

**Keywords:** Stroke, tea, coffee, diet

## Abstract

**Background:**

Stroke is a leading global cause of death and disability. Daily tea/coffee intake is consumed by > 50% of populations and may represent an important population-level exposure. Therefore, it is first essential that we better understand the associations between the tea/coffee intake and stroke.

**Aims:**

This research aims to generate hypotheses about the global associations between tea and coffee intake and stroke. These insights will identify interventions for stroke prevention that can be further explored using alternative study designs.

**Methods:**

INTERSTROKE is a large international matched case–control study of first stroke from 32 countries. Participants were asked “how many cups do you drink each day?” of coffee, green tea, black tea, and other tea. Multivariable conditional logistic regression was used to estimate odds ratios (ORs) and 95% confidence intervals (CIs) for associations between intake and stroke.

**Results:**

We included 13,462 cases and 13,488 controls from INTERSTROKE; mean age was 61.7 (13.4) years and 59.6% (n = 16,010) were male. Overall, 19.4% (n = 5239) did not consume tea/coffee, 47.0% (n = 12,666) consumed tea only, 14.9% (n = 4024) consumed coffee alone, and 18.6% (n = 5021) consumed both, with significant regional variations. After multivariable adjustment, there was no association between low/moderate coffee intake and stroke, but high consumption (> 4/day) was associated with higher odds of all stroke (OR = 1.37 (95% CI = 1.06–1.77)) or ischemic stroke (OR = 1.32 (95% CI = 1.00–1.74)). Tea consumption was associated with lower odds of all (OR = 0.81 (95% CI = 0.69–0.94) for highest intake) or ischemic stroke (OR = 0.81 (95% CI = 0.68–0.98) for highest intake).

**Conclusions:**

High coffee consumption was associated with higher odds of all or ischemic stroke; low–moderate coffee had no association with stroke. In contrast, tea consumption was associated with lower odds of stroke. These associations suggest that individuals consider avoiding high coffee consumption (⩾ five cups/day) to impact future stroke risk.

**Data Access Statement:**

The design and rationale of INTERSTROKE was published previously. Individual participant data, or other documents are not available.

## Introduction

Stroke is a leading cause of death and disability;^1^ modification of common risk factors presents an attractive population-level approach for prevention. While dietary modification is a major target, daily beverage intake may represent an equally important exposure.^2^ In particular, coffee and tea are widely consumed (> 50% of people consuming one or both).^3,4^

The rationale for considering tea and coffee as risk factors for stroke is based on biological and epidemiological evidence. Both frequently contain caffeine, which increases blood pressure (BP,^5^ a leading risk factor), but also contain biologically active constituents (e.g. hydroxycinnamic acids and catechins) that reduce atherosclerosis and impact endothelial function,^6^ insulin resistance, inflammation, and cytokine activation.^7^ Therefore, their potential clinical impacts on stroke are likely to be numerous and it is difficult to estimate net effect from understanding their biological effects alone. Epidemiologically, prospective cohort studies report a null effect or reduced risk of stroke associated with tea^8^ and coffee.^9–12^ However, studies are generally confined to higher-income countries.^13–15^ A key consideration is that beverage consumption may have differing patterns by social setting or population. Therefore, if consistent patterns of association are seen across different populations (with different patterns of confounding), this may reinforce that such associations are more likely to be causal.

## Aims and hypothesis

This research aims to generate hypotheses on the association between tea and coffee intake and stroke, to inform future interventions targeting stroke prevention (in clinical trials). The INTERSTROKE study identified that 10 modifiable risk factors were collectively associated with 90% of the global population-attributable risk of stroke.^16^ This geographically and ethnically diverse study is ideally placed to explore the associations between tea and coffee consumption and stroke.

## Methods

INTERSTROKE is a large international case–control study, published previously.^16^ In brief, cases of first stroke (within 5 days of symptom onset and admitted to hospital within 3 days of presentation) were recruited from 142 centers in 32 countries from March 2007 to July 2015. Recruitment occurred a mean of 2.5 days after stroke symptom onset. Neuroimaging was performed in 99.9% of cases. Information was obtained from the patient or a proxy respondent, if appropriate. Controls, without acute stroke, were recruited and matched to cases for age (< 5 years difference or < 10 years if aged > 90 years), sex, and community/hospital.

Risk factors were assessed through standardized structured questionnaires and physical examination. BP was measured at interview and estimated pre-admission level.^16^ Self-reported items included medical history, physical activity, diet (modified Alternative Health Eating Index (mAHEI)),^17^ smoking and psychological factors.^18^ Hypertension was defined as a history of hypertension or BP ⩾ 140/90 mmHg. Diabetes mellitus was defined as a history of diabetes or HbA1c ⩾ 6.5%. Countries were grouped by geographical region: (1) Western Europe and North America (Canada, Australia, Germany, Denmark, Sweden, United Kingdom, and Ireland); (2) Eastern and Central Europe and Middle East (Croatia, Poland, Turkey, Iran, United Arab Emirates, Russia, and Saudi Arabia); (3) China; (4) South America (Argentina, Brazil, Chile, Colombia, Ecuador, and Peru); (5) Southeast Asia (Thailand, Philippines, and Malaysia); (6) South Asia (India and Pakistan); and (7) Africa (South Africa, Mozambique, Uganda, Sudan, and Nigeria).

Participants were asked one time about their beverage intake (before stroke for cases) “how many cups do you drink each day?” of coffee, Chinese/Japanese green tea, black tea, and other tea. The cup volume was estimated at 250ml. All tea consumption is the sum of the three tea types. Participant intake was categorized as none, one to two cups/day, three to four cups/day, or > four cups/day for each beverage. Participants were asked about adding milk to tea/coffee. Intake of water, fruit juice, and carbonated beverages were collected using a food frequency questionnaire. All data were transferred to Population Health Research Institute, McMaster University and Hamilton Health Sciences, Canada, for quality control. The study was approved by ethics committees in all centers and participants (or proxy) provided written informed consent.

### Statistical analysis

We calculated means and medians to summarize continuous variables, compared by *t*-test, chi-square or appropriate non-parametric tests, including k-sample test on the equality of medians. Correlation coefficient explored the relationship between tea and coffee intake. Conditional logistic regression estimated odds ratios (ORs) and 95% confidence intervals (CIs) for all analyses of associations between beverage intake and all stroke, ischemic stroke and intracerebral hemorrhage (ICH). Multivariable adjustment included age, ethnicity, education, occupation, body mass index (BMI), physical activity, alcohol intake, smoking, diet quality (mAHEI), apoB: apoA, diabetes, hypertension, cardiac risk factors (atrial fibrillation, atrial flutter, rheumatic heart disease), global stress, intake of water and other beverages, adding milk to tea/coffee and the interaction between coffee and tea (all tea (*p*_int_ 0.001), black tea (*p*_int_ 0.003), green tea (*p*_int_ 0.031), or other tea (*p*_int_ 0.038)).

For all stratified or sensitivity analyses, we present associations with all stroke only. We explored whether associations differed by age, sex, smoking, alcohol consumption and hypertension. Differential effects between strata were statistically significant if the *p*-value for the interaction (*p*_int_) between the stratifying variable and beverage was < 0.05. As sensitivity analyses, we restricted to those that completed the questionnaire themselves (to reduce potential bias introduced by proxies).

## Results

We include 13,462 cases and 13,488 of controls from INTERSTROKE, whose characteristics were published previously.^16^ Overall, mean age was 61.7 (13.4) years and 59.6% (n = 16,010) were male. There was representation from seven geographical regions of the world, with the largest representations from China (29.5%) and South Asia (21.3%; see Table 1).

**Table 1.** Characteristics of study population stratified by consumption of tea or coffee.

		Overall	Neither	Tea only	Coffee only	Both tea and coffee	*p*
		n = 26,950	n = 5239	n = 12,666	n = 4024	n = 5021	
Age		61.7 (13.4)	61.4 (13.2)	61.2 (13.2)	61.9 (14.0)	63.4 (13.6)	< 0.001
Male		40.4% (10,894)	50.0% (2621)	36.9% (4677)	38.3% (1539)	41.0% (2057)	< 0.001
Education	< 8 years	48.3% (13,018)	61.0% (3197)	56.1% (7107)	34.9% (1403)	26.1% (1311)	< 0.001
	9–12 years	26.1% (7034)	25.1% (1316)	24.1% (3048)	31.4% (1263)	28.0% (1407)	
	Trade/College/University	25.6% (6892)	13,8% (725)	19.8% (2509)	33.7% (1357)	45.9% (2301)	
Occupation	Skilled/Gen Labor/Farmer	50.6% (13,634)	63.0% (3296)	52.8% (6683)	43.4% (1746)	38.1% (1909)	< 0.001
	Police/Military/Clerical	5.3% (1436)	2.0% (107)	5.8% (728)	5.5% (220)	7.6% (381)	
	Professional/Business	19.7% (5308)	14.6% (764)	17.3% (2189)	18.5% (746)	32.1% (1609)	
	Housewife	16.2% (4366)	14.0% (735)	18.4% (2334)	16.5% (664)	12.6% (633)	
	Disability/Social Security	2.4% (657)	3.8% (199)	1.0% (127)	5.4% (219)	2.2% (112)	
	Other	5.7% (1531)	2.6% (134)	4.7% (597)	10.6% (428)	7.4% (372)	
Smoking	Never	58.7% (15,816)	70.5% (3690)	58.2% (7364)	50.9% (2046)	54.1% (2716)	< 0.001
	Former	14.9% (4025)	7.9% (415)	10.2% (1289)	25.1% (1008)	26.2% (1313)	
	Current	26.3% (7095)	21.6% (1129)	31.7% (4009)	24.1% (969)	19.7% (988)	
Mainly inactive		86.7% (23,339)	94.9% (4968)	90.3% (11,425)	83.6% (3364)	71.4% (3582)	< 0.001
Diet—AHEI tertile	1	35.9% (9679)	34.1% (1786)	36.8% (4662)	41.8% (1682)	30.9% (1549)	< 0.001
	2	33.7% (9074)	41.5% (2174)	33.0% (4181)	29.5% (1185)	30.6% (1534)	
	3	30.4% (8197)	24.4% (1279)	30.2% (3823)	28.8% (1157)	38.6% (1938)	
BMI		25.7 (4.8)	25.0 (4.1)	25.1 (4.8)	26.6 (5.0)	27.1 (4.9)	< 0.001
WHR		0.93 (0.08)	0.91 (0.07)	0.93 (0.08)	0.95 (0.08)	0.94 (0.08)	< 0.001
Hypertension		61.4% (16,553)	58.8% (3078)	59.2% (7503)	68.0% (2736)	64.% (3326)	< 0.001
Diabetes		25.0% (6733)	16.8% (877)	26.8% (3393)	28.7% (1154)	26.1% (1309)	< 0.001
Cardiac risk factors		9.5% (2557)	5.4% (281)	7.8% (989)	12.3% (493)	15.8% (794)	< 0.001
Myocardial infarction		3.5% (940)	1.5% (77)	2.7% (338)	4.7% (187)	6.7% (338)	< 0.001
Atrial fibrillation		3.2% (869)	2.1% (112)	2.3% (290)	4.0% (162)	6.1% (305)	< 0.001
Beverage intake							
Coffee (cups/day), median (IQR)		0 (0–1)	0	0	2 (1–3)	2 (1–2)	< 0.001
All tea (cups/day), median (IQR)		2 (0–3)	0	3 (2–4)	0	2 (1–3)	< 0.001
Black tea (cups/day), median (IQR)		0 (0–0)	0	0 (0–1)	0	1 (0–3)	< 0.001
Green tea (cups/day), median (IQR)		0 (0–0)	0	0 (0–0)	0	0 (0–0)	< 0.001
Other tea (cups/day), median (IQR)		0 (0–2)	0	2 (0–3)	0	0 (0–1)	< 0.001
Water (cups/day), median (IQR)		4 (2–8)	4 (3–6)	4 (2–8)	5 (3–8)	4 (1–7)	< 0.001
Fruit juice (times/week), median (IQR)		0 (0–1)	0 (0–0)	0 (0–1)	1 (1–1)	1 (0–1)	< 0.001
Carbonated beverages (times/week), median (IQR)		0 (0–1)	0 (0–0)	2 (0–3)	0 (0–0)	0 (0–1)	< 0.001
Add milk to tea/coffee		39.0% (15,504)	0	55.4% (7019)	17.9% (720)	52.5% (2637)	< 0.001

AHEI: Alternative Health Eating Index; BMI: body mass index; WHR: waist to hip ratio; IQR: interquartile range.

### Beverage consumption

Overall, 19.4% (n = 5239) had no tea or coffee consumption, 47.0% (n = 12,666) consumed tea only, 14.9% (n = 4024) consumed coffee alone, and 18.6% (n = 5021) consumed both tea and coffee (see Table 1). Non-consumers were younger, less educated, non-smokers, inactive, had lower BMI, and had lower waist to hip ratios (WHRs). They were also less likely to have hypertension, diabetes, myocardial infarction, and atrial fibrillation.

**Figure 1.**
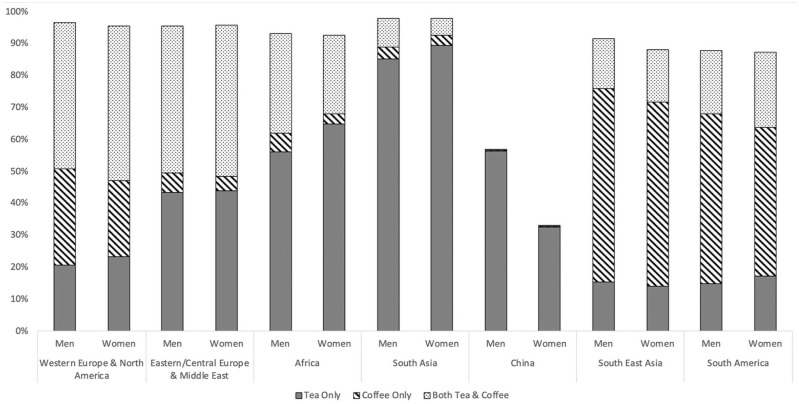
Prevalence of tea and coffee consumption by region. Prevalence by sex, within seven geographical regions. Stacked columns add to 100%, blank area indicates no consumption of tea or coffee.

There were significant variations in tea and coffee consumption by region, with the lowest consumption in China. Within regions, the patterns were broadly similar by sex, other than in China where consumption was lower in women (see Figure 1). There was a weak correlation between coffee and tea (r = 0.1095) overall, but a strong interaction (*p*_int_ < 0.001) for models relating tea and coffee with stroke. Therefore, we did not create a composite for all coffee and tea.

High coffee consumers were younger, female, smokers with lower diet quality, higher BMI and WHR, and resident in Western Europe/North America, South America, or South East Asia (see Supplemental Table S1, Figure S1A). High all tea consumers were older, male, non-smokers with higher activity, diet quality and BMI based in Eastern Central Europe, Western Europe/North America or South Asia (see Supplemental Table S2, Figure S1B). Black tea consumers had higher levels of risk factors (see Supplemental Table S3, Figure S1C); green tea consumers had lower levels of risk factors (see Supplemental Table S4, Figure S1D); and there were few associations between other tea consumption and risk factors (see Supplemental Table S5, Figure S1E).

### Beverage consumption and stroke

**Figure 2.**
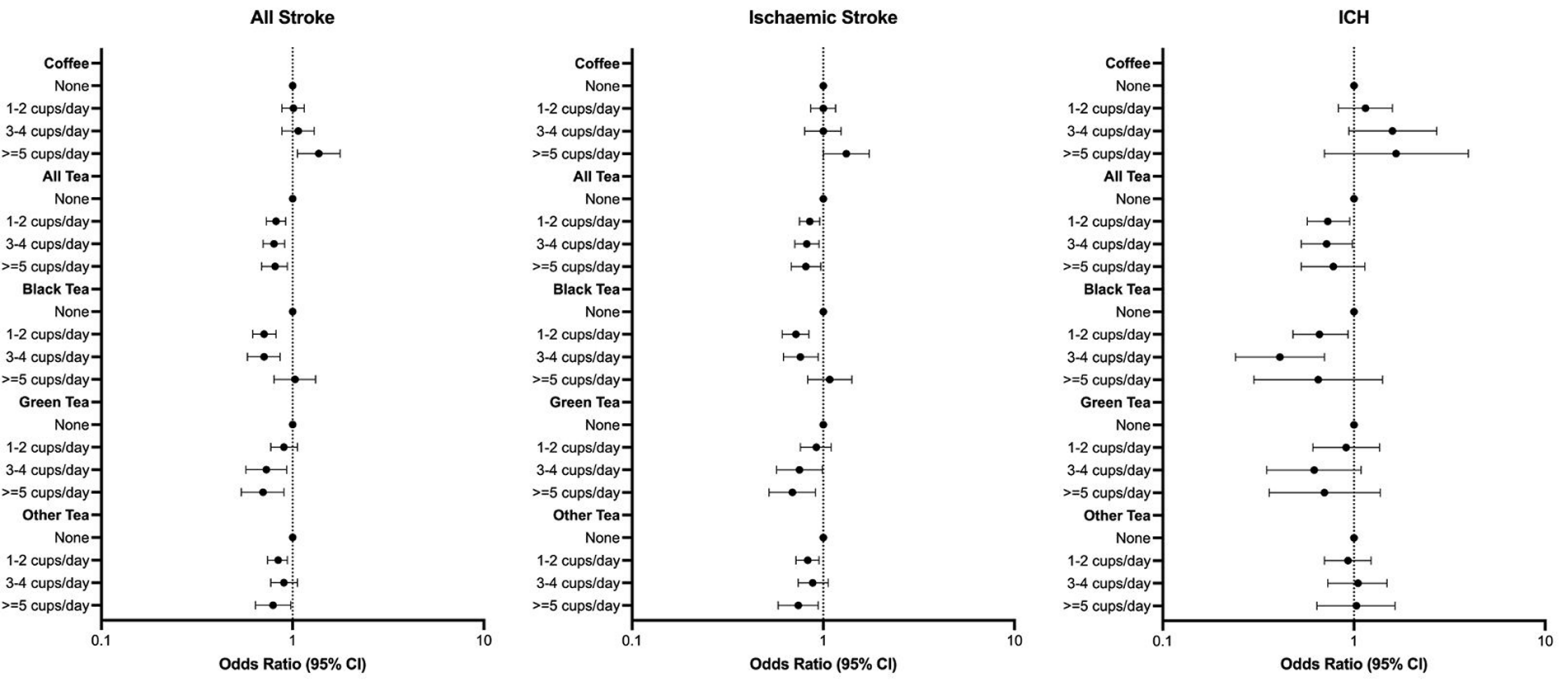
Multivariable adjusted association between tea and coffee intake and stroke. Conditional logistic regression adjusted for age, ethnicity, education, occupation, BMI, physical activity, alcohol, smoking, diet (tertile), apob_apoa, diabetes, hypertension, cardiac risk factors, global stress, coffee, black tea, green tea, other tea, water, fruit drink, carbonated beverage, adding milk to tea or coffee and relevant interaction between tea and coffee.

After multivariable adjustment, there was no association between low/moderate coffee intake and stroke. However, high consumption (> four cups/day) was associated with higher odds of all stroke (OR = 1.37 (95% CI = 1.06–1.77)) and ischemic stroke (OR = 1.32 (95% CI = 1.00–1.74)) but not ICH (see Table 2, Figure 2).

**Table 2.** Associations between tea and coffee intake and stroke.

	None	1–2 cups/day	3–4 cups/day	> 4 cups/day
Coffee				
Cases of all stroke	8973	3074	947	468
Unadjusted	1.00 (Ref)	0.93 (0.86–1.01)	0.98 (0.87–1.10)	1.31 (1.11–1.53)
Adjusted	1.00 (Ref)	1.01 (0.88–1.15)	1.07 (0.88–1.30)	1.37 (1.06–1.77)
Cases of ischemic stroke	6664	2500	814	424
Unadjusted	1.00 (Ref)	0.92 (0.84–1.01)	0.95 (0.84–1.08)	1.32 (1.12–1.57)
Adjusted	1.00 (Ref)	1.00 (0.86–1.16)	1.00 (0.80–1.24)	1.32 (1.00–1.74)
Cases of ICH	2309	574	133	44
Unadjusted	1.00 (Ref)	0.97 (0.80–1.17)	1.15 (0.84–1.56)	1.10 (0.67–1.78)
Adjusted	1.00 (Ref)	1.15 (0.83–1.59)	1.59 (0.94–2.71)	1.66 (0.70–3.96)
All tea				
Cases of all stroke	4854	4280	2890	1438
Unadjusted	1.00 (Ref)	0.76 (0.71–0.82)	0.72 (0.66–0.79)	0.79 (0.72–0.87)
Adjusted	1.00 (Ref)	0.82 (0.73–0.92)	0.80 (0.70–0.91)	0.81 (0.69–0.94)
Cases of ischemic stroke	3660	3268	2246	1228
Unadjusted	1.00 (Ref)	0.79 (0.73–0.86)	0.76 (0.69–0.83)	0.84 (0.75–0.93)
Adjusted	1.00 (Ref)	0.85 (0.75–0.96)	0.82 (0.71–0.95)	0.81 (0.68–0.97)
Cases of ICH	1194	1012	644	210
Unadjusted	1.00 (Ref)	0.67 (0.57–0.78)	0.61 (0.51–0.74)	0.62 (0.49–0.79)
Adjusted	1.00 (Ref)	0.73 (0.57–0.95)	0.72 (0.53–0.98)	0.78 (0.53–1.14)
Black tea				
Cases of all stroke	10,334	1669	991	468
Unadjusted	1.00 (Ref)	0.75 (0.68–0.82)	0.80 (0.71–0.90)	1.00 (0.85–1.17)
Adjusted	1.00 (Ref)	0.71 (0.62–0.82)	0.71 (0.58–0.86)	1.03 (0.80–1.32)
Cases of ischemic stroke	7732	1367	876	427
Unadjusted	1.00 (Ref)	0.78 (0.70–0.86)	0.84 (0.74–0.95)	1.05 (0.89–1.24)
Adjusted	1.00 (Ref)	0.72 (0.61–0.84)	0.76 (0.62–0.94)	1.08 (0.83–1.41)
Cases of ICH	2602	302	115	41
Unadjusted	1.00 (Ref)	0.64 (0.51–0.79)	0.62 (0.45–0.86)	0.70 (0.44–1.12)
Adjusted	1.00 (Ref)	0.66 (0.48–0.93)	0.41 (0.24–0.70)	0.65 (0.30–1.41)
Green tea				
Cases of all stroke	12,202	744	252	264
Unadjusted	1.00 (Ref)	0.89 (0.79–0.99)	0.73 (0.61–0.87)	0.68 (0.56–0.82)
Adjusted	1.00 (Ref)	0.90 (0.77–1.06)	0.73 (0.57–0.93)	0.70 (0.54–0.90)
Cases of ischemic stroke	9372	607	200	223
Unadjusted	1.00 (Ref)	0.90 (0.79–1.02)	0.72 (0.59–0.88)	0.71 (0.58–0.88)
Adjusted	1.00 (Ref)	0.92 (0.76–1.10)	0.75 (0.57–0.99)	0.69 (0.52–0.91)
Cases of ICH	2830	137	52	41
Unadjusted	1.00 (Ref)	0.84 (0.65–1.08)	0.75 (0.50–1.11)	0.55 (0.36–0.85)
Adjusted	1.00 (Ref)	0.91 (0.61–1.36)	0.62 (0.35–1.09)	0.70 (0.36–1.37)
Other tea				
Cases of all stroke	8446	2897	1589	530
Unadjusted	1.00 (Ref)	0.85 (0.79–0.92)	0.86 (0.78–0.95)	1.01 (0.88–1.15)
Adjusted	1.00 (Ref)	0.84 (0.74–0.94)	0.90 (0.77–1.06)	0.79 (0.64–0.98)
Cases of ischemic stroke	6688	2146	1153	415
Unadjusted	1.00 (Ref)	0.86 (0.79–0.94)	0.87 (0.78–0.97)	1.03 (0.88–1.20)
Adjusted	1.00 (Ref)	0.83 (0.72–0.95)	0.88 (0.74–1.06)	0.74 (0.58–0.94)
Cases of ICH	1758	751	436	115
Unadjusted	1.00 (Ref)	0.82 (0.70–0.97)	0.81 (0.66–0.99)	0.93 (0.69–1.25)
Adjusted	1.00 (Ref)	0.93 (0.70–1.23)	1.05 (0.73–1.49)	1.03 (0.64–1.64)

ICH: intracerebral hemorrhage.

Conditional logistic regression models; adjusted for age, ethnicity, education, occupation, body mass index (BMI), physical activity, alcohol, smoking, diet (tertile), apob_apoa, diabetes, hypertension, cardiac risk factors, global stress, other beverage intake (coffee, black tea, green tea, other tea, water, fruit drink and carbonated beverage, as appropriate), adding milk to tea or coffee and the interaction between tea and coffee (all tea/coffee, black tea/coffee, green tea/coffee or othertea/coffee, as appropriate).

All levels of any tea consumption were associated with lower odds of all stroke (OR = 0.81 (95% CI = 0.69–0.94) for highest intake) and ischemic stroke (OR = 0.81 (95% CI = 0.68–0.97) for highest intake); only low–moderate intake was associated with lower odds of ICH (OR = 0.72 (95% CI = 0.53–0.98 for three to four cups/day). Low–moderate black tea consumption was associated with lower odds of all stroke (OR = 0.71 (95% CI = 0.58–0.86) for three to four cups/day), ischemic stroke (OR = 0.76 (95% CI = 0.62–0.94) for three to four cups/day), and ICH (OR = 0.41 (95% CI 0.24–0.70) for three to four cups/day). Moderate–high green tea consumption was associated with lower odds of all stroke (OR = 0.70 (95% CI = 0.54–0.90) for highest intake) and ischemic stroke (OR = 0.69 (95% CI = 0.52–0.91) for highest intake) but not ICH. Both low and high consumption of other tea was associated with lower odds of all stroke (OR = 0.79 (95% CI = 0.64–0.98) for highest intake) and ischemic stroke (OR = 0.74 (95% CI = 0.58–0.94) for highest intake).

There were significant differences in these associations by region (see Supplemental Table S6). For coffee, there were lower odds of all stroke only in Western Europe/North America (OR = 0.71 (95% CI = 0.52–0.97); *p*_int_ = 0.048). For tea, there were lower odds of all stroke only in China (OR = 0.77 (95% CI = 0.64–0.93)) and South America (OR = 0.58 (95% CI = 0.43–0.78)) and higher odds of all stroke in South Asia (OR = 2.20 (95% CI = 1.19–4.07); *p*_int_ = 0.001).

### Subgroup and sensitivity analyses

The associations between tea/coffee consumption and stroke by age (see Supplemental Table S7), sex (see Supplemental Table S8), and smoking (see Supplemental Table S9) were consistent. For alcohol consumption, there were higher odds of all stroke for never/former drinkers consuming three to four or more than four cups of coffee per day (*p*_int_ < 0.001; see Supplemental Table S10). There were statistically significant differences by hypertension status, for coffee (*p*_int_ 0.001), all tea (*p*_int_ 0.03), and black tea (*p*_int_ 0.003; see Supplemental Table S11). The addition of milk to coffee did not significantly alter associations (*p*_int_ 0.07), but the lower odds of stroke associated with tea consumption was restricted to those that did not add milk (all tea *p*_int_ < 0.01, black tea *p*_int_ 0.03, and other tea *p*_int_ < 0.01; see Supplemental Table S12). Associations between beverage intake and all stroke were similar on sensitivity analyses that excluded proxy respondents (see Supplemental Table S13).

## Discussion

In this large international study, we report considerable variations in tea and coffee consumption, and characteristics of consumers among across the world. High coffee consumption was associated with higher odds of all stroke and ischemic stroke, but not ICH (although power was limited). There was no association between low–moderate coffee intake and stroke. Tea consumption (black, green, or other) was associated with lower odds of stroke. There were important regional variations; coffee consumption was associated with lower odds of stroke in Western Europe/North America only and tea consumption was associated with lower odds of stroke in China and South America. Tea consumption was associated with higher odds of stroke in South Asia.

Coffee and tea are among the most widely consumed beverages; therefore, small effects could have large public health impacts. Although both usually contain caffeine, which increases peripheral vascular resistance and BP,^5^ they also contain biologically active constituents (e.g. epigallocatechin gallate) with antioxidant properties that reduce cardiometabolic risk.^19^ Therefore, it is not surprising that we report conflicting patterns of association between coffee and tea, with respect to stroke. In addition, similar to other reports,^13^ we identified a correlation between coffee and tea, and a significant interaction between the two exposures and stroke.

We observed that frequent coffee consumers had higher cardiovascular risk burdens, contrasting with previous reports that coffee is associated with lower body weight and cholesterol.^20^ Despite adjusting for these risk factors, high coffee consumption remained associated with higher odds of all stroke and ischemic stroke, contrasting with two recent meta-analyses where coffee consumption was associated with reduced stroke risk.^9,21^ We also observed a statistically significant interaction for coffee intake by hypertension status, with a trend toward increased higher odds of stroke in those with hypertension, although confidence intervals overlapped broadly.

We report important regional variations, with lower odds of all stroke in Western Europe and North America only. This may reflect regional, cultural, or social factors underpinning coffee preparation and consumption including the coffee bean (Arabica vs. Robusta), roasting (vs. unroasted), preparation method and brew type,^22^ which may alter bioactive compounds. Although coffee contains more antioxidants than tea, roasting may degrade abundant antioxidants.^23^ Our findings from Western Europe and North America are consistent with previous reports,^13,24,25^ where coffee intake was associated with reduced risk of stroke, similar to a recent meta-analysis.^9^ We report no significant differences by sex, unlike a Korean study that reported an inverse association between coffee intake and stroke in females only.^26^ The effects of coffee within an individual may vary by gut microbiome;^27^ perhaps this explains our observations in never/former drinkers.

In contrast to coffee, frequent tea consumers were non-smokers with higher activity and diet quality. Tea consumption was associated with lower odds of all stroke, ischemic stroke and ICH. The greatest magnitude of association was seen for consumption of three to four cups/day of black tea for ICH. Similar to coffee intake, we observed a statistically significant interaction for all tea or black tea intake by hypertension status, with a trend toward increased lower odds of stroke in those with hypertension, although confidence intervals overlapped broadly.

Tea, originating from *Camellia sinensis*, can be consumed as black (fully oxidized), green (non-oxidized), or oolong (partially oxidized).^28^ The oxidation process impacts caffeine, polyphenols, and flavonoids with antioxidative, anti-inflammatory, and anti-apoptotic effects.^29^ Green tea contains catechins with important antioxidative,^30^ anti-inflammatory,^31^ and anti-thrombogenic mechanisms^28^ that alleviate atherosclerosis,^32^ ameliorate ischemia/reperfusion injury, and enhance endothelial function.^33^ These mechanisms likely explain our observed reduction in odds of stroke with tea, consistent with other studies.^8,13,24^ Geographical and cultural factors also impact tea consumption, particularly green or other tea. Importantly, black tea consumers had higher cardiovascular risk factor burdens than green tea consumers. This may explain our regional differences including lower odds of all stroke in China (consistent with previous studies)^34^ and South America. We report higher odds of all stroke only in South Asia. Although we adjusted for the addition of milk to tea and coffee, we did not have data on the addition of important items including sugar, syrups, spices, or other items, which may impact or counteract the potentially beneficial constituents of tea, or directly increase stroke risk.

The major strengths of this study are the large number of individuals from a board range of countries, regions, and ethnicities, which allowed us to determine significant regional variations in the coffee and tea (including black, green, and other subtypes). The case–control design provided a practical approach to achieving a level of diversity and ensuring global representation, including populations generally excluded from previous studies. In addition, while there may be significant differences between countries (e.g. social deprivation, culture, diet, or lifestyle habits), our matched case–control design, where controls were recruited from the same center/country, reduces the impact of these factors. We have a large volume of data, with the ability to control for many covariates to minimize the effects of confounding.

Our study has potential limitations. As it is a case–control study, it may be vulnerable to recall bias, particularly if there is differential recollection of beverage intake between cases and controls, or exacerbated where the questionnaire was completed by or with the assistance of a proxy (e.g. more severe neurological deficits). Therefore, we completed sensitivity analyses restricted to responses exclusively provided by participants, and our observations were unchanged. Cases were also recruited shortly after stroke onset, which reduces the risk of recall bias. However, cases may recall their intake differently than controls, if cases are more likely to consider that previous lifestyle or behaviors contributed to stroke. In addition, this may differ across regions due to variations in the prevalence of beverage consumption, health promotion, and public health strategies. We do not have data to support this exploration. Selection bias may have resulted from the approach to recruiting controls, but we reduced this by excluding those with a hospital referral or diagnosis related to stroke. In addition, data were collected in a standardized manner, identical in cases and controls. Causality cannot be firmly established and although we adjusted for multiple confounders, our findings may be influenced by residual confounding or unmeasured confounders. We had limited numbers of cases of stroke within subtypes of ischemic stroke; therefore, we chose not to present data by subtype, although a recent study reported that tea consumption was associated with reduced odds of small vessel stroke.^35^ Tea and coffee consumption was measured at one time point only and is unlikely to represent long-term or cumulative lifetime exposure. We do not have data on the caffeine content (caffeinated vs decaffeinated beverages) on the addition of sugar or syrups, to tea or coffee, which holds the potential to impact cardiovascular risk; however, we were able to adjust for the addition of milk. Although the addition of milk did not alter associations with coffee, the lower odds of stroke associated with all tea, black tea, or other tea consumption were restricted to those that did not add milk. Importantly, we are not aware of specific resources with robust global data on additions, other than milk, to estimate the extent of their impact. In addition, we do not have important data on characteristics of coffee including preparation/brewing method, type of coffee consumed (e.g. instant coffee vs espresso, light vs dark roasted coffee beans) or addition of non-milk preparations, which may also alter the level of antioxidants. Our study was confined to those who survived long enough to reach a hospital, rather than all individuals with stroke and we could not include the most severe (fatal) strokes.

## Conclusion

We report that only high coffee consumption is associated with higher odds of all stroke and ischemic stroke; low–moderate coffee intake was not associated with stroke. In contrast, tea consumption was associated with lower odds of stroke and no level of tea consumption was associated with higher odds of stroke. While there were important regional variations (e.g. coffee consumption was associated with lower odds of stroke in Western Europe/North America), we suggest that individuals consider avoiding very high levels of coffee consumption (five or more cups/day) and to reduce or eliminate the addition of milk to tea in order to impact future stroke risk.

## Supplemental Material

sj-docx-1-wso-10.1177_17474930241264685 – Supplemental material for Tea and coffee consumption and risk of acute stroke: The INTERSTROKE Study Supplemental material, sj-docx-1-wso-10.1177_17474930241264685 for Tea and coffee consumption and risk of acute stroke: The INTERSTROKE Study by Andrew Smyth, Graeme J Hankey, Peter Langhorne, Catriona Reddin, Danuta Ryglewicz, Annika Rosengren, Dennis Xavier, Michelle Canavan, Shahram Oveisgharan, Xingyu Wang, Patricio Lopez Jaramillo, Albertino Damasceno, Anna Czlonkowska, Helle Klingenberg Iversen, Fernando Lanas, Salim Yusuf and Martin O’Donnell in International Journal of Stroke
