# Supplementary material for: Tea and coffee consumption and risk of acute stroke: The INTERSTROKE Study
Source: Int J Stroke. 2024 Jul 31;19(9):1053–63. doi: 10.1177/17474930241264685 (PMC11523547; doi:10.1177/17474930241264685)
Supplement: sj-docx-1-wso-10.1177_17474930241264685 – Supplemental material for Tea and coffee consumption and risk of acute stroke: The INTERSTROKE Study [file sj-docx-1-wso-10.1177_17474930241264685.docx]

**Supplementary Materials**

Figure S1. Level of Intake of Tea or Coffee by Region

Table S1. Population Characteristics Stratified by Coffee Consumption

Table S2. Population Characteristics Stratified by Any Tea Consumption

Table S3. Population Characteristics Stratified by Black Tea Consumption

Table S4. Population Characteristics Stratified by Green Tea Consumption

Table S5. Population Characteristics Stratified by Other Tea Consumption

Table S6. Association Between Tea/Coffee and All Stroke by Region

Table S7. Association between Tea/Coffee Consumption and All Stroke – Stratified by Age

Table S8. Association between Tea/Coffee Consumption and All Stroke – Stratified by Sex

Table S9. Association between Tea/Coffee Consumption and All Stroke – Stratified by Smoking

Table S10. Association between Tea/Coffee Consumption and All Stroke – Stratified by Alcohol Consumption

Table S11. Association between Tea/Coffee Consumption and All Stroke – Stratified by Hypertension Status

Table S12. Association between Tea/Coffee Consumption and All Stroke – Stratified by Addition of Milk to Tea/Coffee

Table S13. Association between Tea/Coffee Consumption and All Stroke Excluding Proxy Assistance

**Figure S1. Level of Intake of Tea or Coffee by Region**

1. Coffee
2. All Types of Tea
3. Black Tea
4. Green Tea
5. Other Tea

**Table S1. Population Characteristics Stratified by Coffee Consumption**

|  | | **None** | **1-2 cups/day** | **3-4 cups/day** | **>4 cups/day** | **p** |
| --- | --- | --- | --- | --- | --- | --- |
|  |  | **n=17,905** | **n=6,310** | **n=1,908** | **n=827** |  |
| Age | | 61.2 (13.2) | 63.2 (14.1) | 62.4 (13.2) | 60.3 (12.4) | <0.001 |
| Male | | 59.2% (10,607) | 57.7% (3,639) | 35.3% (673) | 30.5% (252) | <0.001 |
| Education | <8 years | 57.6% (10,304) | 31.6% (1,993) | 26.3% (501) | 26.6% (220) | <0.001 |
|  | 9-12 years | 24.4% (4,364) | 29.5% (1,858) | 28.1% (537) | 33.3% (275) |  |
|  | Trade/College/University | 18.1% (3,234) | 38.9% (2,456) | 45.6% (870) | 40.2% (332) |  |
| Occupation | Skilled/Gen Labour/Farmer | 55.8% (9,979) | 40.4% (2,548) | 39.2% (748) | 43.4% (359) | <0.001 |
|  | Police/Military/Clerical | 4.7% (835) | 6.5% (410) | 7.7% (146) | 5.4% (45) |  |
|  | Professional/Business | 16.5% (2,953) | 25.5% (1,609) | 28.5% (544) | 24.4% (202) |  |
|  | Housewife | 17.2% (3,069) | 16.8% (1,061) | 9.1% (174) | 7.5% (62) |  |
|  | Disability/Social Security | 1.8% (326) | 3.3% (208) | 4.4% (83) | 4.8% (40) |  |
|  | Other | 4.1% (731) | 7.5% (470) | 11.1% (211) | 14.4% (119) |  |
| Smoking | Never | 61.8% (11,054) | 57.9% (3,652) | 46.0% (877) | 28.2% (233) | <0.001 |
|  | Former | 9.5% (1,704) | 24.1% (1,518) | 29.4% (561) | 29.3% (242) |  |
|  | Current | 28.7% (5,138) | 18.0% (1,136) | 42.6% (469) | 42.6% (352) |  |
| Mainly Inactive | | 91.6% (16,393) | 79.5% (5,017) | 69.3% (1323) | 73.4% (606) | <0.001 |
| Diet – AHEI Tertile | 1 | 36.0% (6,448) | 34.7% (2,190) | 35.4% (675) | 44.3% (366) | <0.001 |
|  | 2 | 35.5% (6,355) | 30.8% (1,942) | 29.6% (564) | 25.8% (213) |  |
|  | 3 | 28.5% (5,102) | 34.5% (2,178) | 35.1% (669) | 30.0% (248) |  |
| BMI | | 25.1 (4.6) | 26.8 (4.9) | 27.2 (5.1) | 27.1 (5.0) | <0.001 |
| WHR | | 0.92 (0.07) | 0.94 (0.08) | 0.95 (0.08) | 0.95 (0.09) | <0.001 |
| Hypertension | | 59.1% (10,581) | 67.1% (4,236) | 65.4% (1,248) | 59.0% (488) | <0.001 |
| Diabetes | | 23.9% (4,270) | 28.2% (1,779) | 26.5% (505) | 21.6% (179) | <0.001 |
| Cardiac Risk Factors | | 7.1% (1,270) | 14.4% (906) | 14.2% (270) | 13.4% (111) | <0.001 |
| Myocardial Infarction | | 2.3% (415) | 5.7% (357) | 6.1% (116) | 6.3% (52) | <0.001 |
| Atrial Fibrillation | | 2.3% (402) | 5.1% (319) | 5.2% (99) | 5.9% (49) | <0.001 |

**Table S2. Population Characteristics Stratified by Any Tea Consumption**

|  | | **None** | **1-2 cups/day** | **3-4 cups/day** | **>4 cups/day** | **p** |
| --- | --- | --- | --- | --- | --- | --- |
|  |  | **n=9,263** | **n=8,785** | **n=6,009** | **n=2,893** |  |
| Age | | 61.6 (13.5) | 61.6 (13.9) | 61.8 (13.0) | 62.2 (12.6) | 0.12 |
| Male | | 55.1% (5,103) | 57.9% (5,083) | 64.6% (3,881) | 68.8% (1,989) | <0.001 |
| Education | <8 years | 49.7% (4,600) | 50.7% (4,455) | 47.8% (2,869) | 37.8% (1,094) | <0.001 |
|  | 9-12 years | 27.9% (2,579) | 23.6% (2,073) | 25.3% (1,522) | 29.7% (860) |  |
|  | Trade/College/University | 22.5% (2,082) | 25.7% (2,253) | 26.9% (1,618) | 32.5% (939) |  |
| Occupation | Skilled/Gen Labour/Farmer | 54.5% (5,042) | 47.6% (4,174) | 50.2% (3,014) | 48.6% (1,404) | <0.001 |
|  | Police/Military/Clerical | 3.5% (327) | 5.4% (474) | 7.1% (426) | 7.2% (209) |  |
|  | Professional/Business | 16.3% (1,510) | 19.5% (1,712) | 21.7% (1,302) | 27.1% (784) |  |
|  | Housewife | 15.1% (1,399) | 20.6% (1804) | 15.3% (918) | 8.5% (245) |  |
|  | Disability/Social Security | 4.5% (418) | 1.4% (120) | 1.2% (71) | 1.7% (48) |  |
|  | Other | 6.1% (562) | 5.6% (491) | 4.6% (276) | 7.0% (202) |  |
| Smoking | Never | 62.0% (5,736) | 61.5% (5,403) | 55.8% (3,350) | 45.9% (1,327) | <0.001 |
|  | Former | 15.4% (1,423) | 14.1% (1,240) | 13.3% (800) | 19.4% (562) |  |
|  | Current | 22.7% (2,098) | 24.4% (2,138) | 30.9% (1,856) | 34.7% (1,003) |  |
| Mainly Inactive | | 90.0% (8,332) | 87.4% (767) | 83.5% (5,016) | 80.0% (2,315) | <0.001 |
| Diet – AHEI Tertile | 1 | 37.4% (3,468) | 37.9% (3,332) | 35.2% (2,114) | 26.4% (765) | <0.001 |
|  | 2 | 36.3% (3,359) | 30.2% (2,654) | 34.4% (2,069) | 34.3% (992) |  |
|  | 3 | 26.3% (2,436) | 31.9% (2,799) | 30.4% (1,826) | 39.3% (1,136) |  |
| BMI | | 25.7 (4.6) | 25.5 (5.0) | 25.6 (4.9) | 26.5 (4.8) | <0.001 |
| WHR | | 0.93 (0.08) | 0.93 (0.08) | 0.93 (0.08) | 0.93 (0.08) | 0.02 |
| Hypertension | | 62.8% (5,814) | 61.4% (5,394) | 59.3% (3,565) | 61.5% (1,780) | <0.001 |
| Diabetes | | 21.9% (2,031) | 27.9% (2,448) | 26.5% (1,592) | 22.9% (662) | <0.001 |
| Cardiac Risk Factors | | 8.4% (774) | 10.1% (883) | 9.0% (542) | 12.4% (358) | <0.001 |
| Myocardial Infarction | | 2.9% (264) | 3.6% (313) | 3.6% (215) | 5.1% (148) | <0.001 |
| Atrial Fibrillation | | 3.0% (274) | 3.2% (278) | 3.0% (178) | 4.8% (139) | <0.001 |

**Table S3. Population Characteristics Stratified by Black Tea Consumption**

|  | | **None** | **1-2 cups/day** | **3-4 cups/day** | **>4 cups/day** | **p** |
| --- | --- | --- | --- | --- | --- | --- |
|  |  | **n=20418** | **n=3,598** | **n=2,047** | **n=887** |  |
| Age | | 61.3 (13.3) | 62.2 (14.3) | 64.4 (13.0) | 63.9 (13.3) | <0.001 |
| Male | | 60.1% (12,271) | 57.3% (2,063) | 56.7% (1,164) | 62.9% (558) | <0.001 |
| Education | <8 years | 51.4% (10,488) | 43.8% (1,577) | 32.2% (659) | 33.2% (294) | <0.001 |
|  | 9-12 years | 26.3% (5,372) | 42.2% (871) | 23.8% (488) | 34.2% (303) |  |
|  | Trade/College/University | 22.3% (4,553) | 31.9% (1,149) | 44.0% (900) | 32.6% (290) |  |
| Occupation | Skilled/Gen Labour/Farmer | 53.1% (10,825) | 43.2% (1,553) | 43.8% (896) | 40.6% (360) | <0.001 |
|  | Police/Military/Clerical | 5.0% (1,024) | 5.4% (194) | 6.5% (132) | 9.7% (86) |  |
|  | Professional/Business | 17.2% (3,505) | 26.5% (954) | 29.9% (611) | 26.9% (238) |  |
|  | Housewife | 16.9% (3,450) | 15.9% (573) | 12.5% (255) | 9.9% (88) |  |
|  | Disability/Social Security | 2.7% (556) | 1.6% (59) | 1.4% (29) | 1.5% (13) |  |
|  | Other | 5.1% (1,045) | 7.3% (261) | 6.1% (124) | 11.4% (101) |  |
| Smoking | Never | 58.7% (11,986) | 61.4% (2,209) | 60.3% (1,234) | 43.6% (387) | <0.001 |
|  | Former | 12.7% (2,588) | 20.3% (730) | 22.5% (461) | 27.7% (246) |  |
|  | Current | 28.6% (5,831) | 18.3% (659) | 17.2% (3,51) | 28.6% (254) |  |
| Mainly Inactive | | 89.7% (18,300) | 80.4% (2,889) | 72.5% (1,482) | 75.3% (668) | <0.001 |
| Diet – AHEI Tertile | 1 | 37.5% (7,652) | 31.5% (1,132) | 31.5% (644) | 28.3% (251) | <0.001 |
|  | 2 | 34.0% (6,945) | 32.4% (1,165) | 33.5% (686) | 31.3% (278) |  |
|  | 3 | 28.5% (5,821) | 36.2% (1,301) | 35.0% (717) | 40.4% (358) |  |
| BMI | | 25.3 (4.7) | 26.6 (5.1) | 27.1 (4.9) | 27.3 (5.2) | <0.001 |
| WHR | | 0.93 (0.07) | 0.93 (0.08) | 0.93 (0.08) | 0.94 (0.09) | <0.001 |
| Hypertension | | 60.8% (12,405) | 63.3% (2,277) | 65.2% (1,334) | 60.5% (537) | <0.001 |
| Diabetes | | 24.9% (5,076) | 26.5% (954) | 23.3% (477) | 25.5% (226) | 0.05 |
| Cardiac Risk Factors | | 7.7% (1,574) | 13.5% (486) | 16.2% (332) | 18.6% (165) | <0.001 |
| Myocardial Infarction | | 2.8% (561) | 4.8% (182) | 6.5% (133) | 8.3% (74) | <0.001 |
| Atrial Fibrillation | | 2.5% (508) | 4.8% (171) | 6.2% (127) | 7.1% (63) | <0.001 |

**Table S4. Population Characteristics Stratified by Green Tea Consumption**

|  | | **None** | **1-2 cups/day** | **3-4 cups/day** | **>4 cups/day** | **p** |
| --- | --- | --- | --- | --- | --- | --- |
|  |  | **n=24,240** | **n=1,543** | **n=566** | **n=601** |  |
| Age | | 61.7 (13.5) | 62.2 (13.1) | 62.6 (11.6) | 61.2 (11.6) | <0.001 |
| Male | | 58.9% (14,282) | 56.9% (878) | 70.0% (396) | 83.2% (500) | <0.001 |
| Education | <8 years | 49.5% (12,006) | 30.4% (469) | 43.5% (246) | 49.4% (297) | <0.001 |
|  | 9-12 years | 26.2% (6,355) | 24.4% (377) | 24.0% (136) | 27.6% (166) |  |
|  | Trade/College/University | 24.2% (5,873) | 45.2% (697) | 32.5% (184) | 23.0% (138) |  |
| Occupation | Skilled/Gen Labour/Farmer | 50.3% (12,175) | 47.2% (728) | 62.2% (352) | 63.1% (379) | <0.001 |
|  | Police/Military/Clerical | 5.5% (1,328) | 4.6% (71) | 2.8% (16) | 3.5% (21) |  |
|  | Professional/Business | 18.6% (4,516) | 31.0% (478) | 25.8% (146) | 28.0% (168) |  |
|  | Housewife | 17.1% (4,150) | 10.8% (167) | 6.0% (34) | 2.5% (15) |  |
|  | Disability/Social Security | 2.6% (633) | 1.1% (17) | 0.4% (2) | 0.8% (5) |  |
|  | Other | 5.9% (1,420) | 5.3% (82) | 2.8% (16) | 2.2% (13) |  |
| Smoking | Never | 59.6% (14,441) | 54.5% (841) | 52.5% (297) | 39.4% (237) | <0.001 |
|  | Former | 14.9% (3,602) | 18.4% (284) | 13.4% (76) | 10.5% (63) |  |
|  | Current | 25.5% (6,183) | 27.1% (418) | 34.1% (193) | 50.1% (301) |  |
| Mainly Inactive | | 87.1% (21,090) | 79.7% (1230) | 83.8% (474) | 90.7% (545) | <0.001 |
| Diet – AHEI Tertile | 1 | 37.4% (9,060) | 27.7% (428) | 19.4% (110) | 13.5% (81) | <0.001 |
|  | 2 | 33.3% (8,066) | 34.9% (538) | 38.9% (220) | 41.6% (250) |  |
|  | 3 | 29.3% (7,114) | 37.4% (577) | 41.7% (236) | 44.9% (270) |  |
| BMI | | 25.7 (4.9) | 26.1 (4.4) | 25.7 (3.8) | 25.5 (3.3) | <0.001 |
| WHR | | 0.93 (0.08) | 0.92 (0.08) | 0.93 (0.06) | 0.94 (0.06) | <0.001 |
| Hypertension | | 61.5% (14,895) | 65.5% (1,010) | 56.5% (320) | 54.5% (328) | <0.001 |
| Diabetes | | 25.6% (6,190) | 22.1% (340) | 18.2% (103) | 16.6% (100) | <0.001 |
| Cardiac Risk Factors | | 9.6% (2,315) | 12.1% (187) | 5.5% (31) | 4.0% (24) | <0.001 |
| Myocardial Infarction | | 3.5% (856) | 4.2% (65) | 1.8% (10) | 1.5% (9) | 0.002 |
| Atrial Fibrillation | | 3.2% (765) | 5.7% (88) | 2.1% (12) | 0.7% (4) | <0.001 |

**Table S5. Population Characteristics Stratified by Other Tea Consumption**

|  | | **None** | **1-2 cups/day** | **3-4 cups/day** | **>4 cups/day** | **p** |
| --- | --- | --- | --- | --- | --- | --- |
|  |  | **n=16,692** | **n=5988** | **n=3,255** | **n=1,015** |  |
| Age | | 62.2 (13.6) | 61.4 (13.6) | 60.6 (12.6) | 60.5 (12.6) | <0.001 |
| Male | | 58.1% (9,700) | 57.2% (3,425) | 67.1% (2,185) | 73.5% (746) | <0.001 |
| Education | <8 years | 45.7% (7,619) | 52.6% (3,149) | 55.6% (1,808) | 43.6% (442) | <0.001 |
|  | 9-12 years | 26.7% (4,451) | 24.1% (1,442) | 25.4% (828) | 30.8% (313) |  |
|  | Trade/College/University | 27.7% (4,620) | 23.3% (1,393) | 19.0% (619) | 25.6% (260) |  |
| Occupation | Skilled/Gen Labour/Farmer | 51.2% (8,537) | 49.0% (2,929) | 51.6% (1,678) | 48.3% (490) | <0.001 |
|  | Police/Military/Clerical | 4.4% (734) | 5.9% (353) | 8.1% (264) | 8.4% (85) |  |
|  | Professional/Business | 21.1% (3,512) | 16.8% (1003) | 17.4% (566) | 22.4% (227) |  |
|  | Housewife | 13.8% (2,309) | 22.8% (1,364) | 17.8% (580) | 11.1% (113) |  |
|  | Disability/Social Security | 3.1% (524) | 1.2% (69) | 1.2% (39) | 2.5% (25) |  |
|  | Other | 6.4% (1,066) | 4.4% (264) | 3.9% (126) | 7.4% (75) |  |
| Smoking | Never | 59.6% (9,942) | 60.9% (3,644) | 54.1% (1,761) | 46.3% (469) | <0.001 |
|  | Former | 17.3% (2,893) | 11.6% (694) | 8.6% (280) | 15.6% (158) |  |
|  | Current | 23.1% (3,850) | 27.5% (1,646) | 37.3% (1,212) | 38.2% (387) |  |
| Mainly Inactive | | 85.5% (14,252) | 90.0% (5,387) | 87.4% (2,843) | 84.4% (857) | <0.001 |
| Diet – AHEI Tertile | 1 | 33.7% (5,620) | 40.5% (2,422) | 39.6% (1,288) | 34.4% (349) | <0.001 |
|  | 2 | 35.4% (5,909) | 28.8% (1,723) | 33.9% (1,104) | 33.3% (338) |  |
|  | 3 | 30.9% (5,163) | 30.8% (1,843) | 26.5% (863) | 32.3% (328) |  |
| BMI | | 26.1 (4.7) | 25.0 (4.8) | 24.8 (5.0) | 25.8 (4.9) | 0.002 |
| WHR | | 0.93 (0.08) | 0.92 (0.08) | 0.93 (0.08) | 0.93 (0.09) | <0.001 |
| Hypertension | | 62.4% (10,423) | 60.8% (3,643) | 57.5% (1,872) | 60.6% (615) | <0.001 |
| Diabetes | | 22.6% (3,775) | 29.2% (1,743) | 29.9% (974) | 23.7% (241) | <0.001 |
| Cardiac Risk Factors | | 10.4% (1,734) | 9.0% (536) | 6.1% (199) | 8.7% (88) | <0.001 |
| Myocardial Infarction | | 3.8% (639) | 3.0% (181) | 2.6% (84) | 3.6% (36) | 0.001 |
| Atrial Fibrillation | | 3.1% (609) | 3.0% (178) | 1.8% (60) | 2.2% (22) | <0.001 |

**Table S6. Association Between Tea/Coffee and All Stroke by Region**

|  | | Western Europe,  North America | East/Central Europe, Middle East | Africa | South Asia | China | South East Asia | South America | p_int_ |
| --- | --- | --- | --- | --- | --- | --- | --- | --- | --- |
|  |  | OR (95% CI) | OR (95% CI) | OR (95% CI) | OR (95% CI) | OR (95% CI) | OR (95% CI) | OR (95% CI) |  |
| **Coffee** | | | | | | | | | |
| *No Coffee* | *Cases* | *555* | *680* | *642* | *2,542* | *3,970* | *206* | *378* | 0.048 |
|  | OR  (95% CI) | 1.00  (Ref) | 1.00  (Ref) | 1.00  (Ref) | 1.00  (Ref) | 1.00  (Ref) | 1.00  (Ref) | 1.00  (Ref) |  |
| *Any Coffee* | *Cases* | *1,362* | *714* | *331* | *323* | *17* | *649* | *1,093* |  |
|  | OR  (95% CI) | 0.71  (0.52-0.97) | 1.22  (0.84-1.76) | 1.53  (0.85-2.75) | 1.34  (0.78-2.29) | 1.61  (0.55-4.76) | 1.17  (0.74-1.83) | 1.01  (0.77-1.33) |  |
| **All Tea** | | | | | | | | | |
| *No Tea* | *Cases* | *653* | *144* | *111* | *166* | *2,186* | *585* | *1,009* | 0.001 |
|  | OR  (95% CI) | 1.00  (Ref) | 1.00  (Ref) | 1.00  (Ref) | 1.00  (Ref) | 1.00  (Ref) | 1.00  (Ref) | 1.00  (Ref) |  |
| *Any Tea* | *Cases* | *1,264* | *1,250* | *862* | *2,699* | *1,801* | *270* | *462* |  |
|  | OR  (95% CI) | 0.78  (0.58-1.05) | 0.89  (0.58-1.36) | 1.45  (0.83-2.55) | 2.20  (1.19-4.07) | 0.77  (0.64-0.93) | 1.22  (0.73-2.04) | 0.58  (0.43-0.78) |  |

Conditional logistic regression models; Adjusted for age, ethnicity, education, occupation, BMI, physical activity, alcohol, smoking, diet (tertile), apob_apoa, diabetes, hypertension, cardiac risk factors, global stress, other beverage intake (coffee or all tea, as appropriate), water, fruit drink and carbonated beverage, adding milk to tea or coffee and the interaction between tea and coffee (all tea/coffee)

**Table S7. Association between Tea/Coffee Consumption and All Stroke – Stratified by Age**

|  | **None** | **1-2cups/day** | **3-4 cups/day** | **>4cups/day** | **p_int_** |
| --- | --- | --- | --- | --- | --- |
| **Coffee** | | | | | |
| Overall | 1.00 (Ref) | 1.01 (0.88-1.15) | 1.07 (0.88-1.30) | 1.37 (1.06-1.77) | - |
| <65years | 1.00 (Ref) | 0.91 (0.76-1.10) | 0.98 (0.75-1.29) | 1.38 (0.96-1.99) | 0.30 |
| >=65 years | 1.00 (Ref) | 1.20 (0.98-1.49) | 1.27 (0.93-1.74) | 1.52 (1.00-2.33) |  |
| **All Tea** | | | | | |
| Overall | 1.00 (Ref) | 0.82 (0.73-0.92) | 0.80 (0.70-0.91) | 0.81 (0.69-0.94) | - |
| <65years | 1.00 (Ref) | 0.73 (0.63-0.86) | 0.73 (0.60-0.87) | 0.72 (0.58-0.89) | 0.80 |
| >=65 years | 1.00 (Ref) | 0.92 (0.77-1.09) | 0.91 (0.73-1.12) | 0.92 (0.71-1.20) |  |
| **Black Tea** | | | | | |
| Overall | 1.00 (Ref) | 0.71 (0.62-0.82) | 0.71 (0.58-0.86) | 1.03 (0.80-1.32) | - |
| <65years | 1.00 (Ref) | 0.67 (0.55-0.82) | 0.72 (0.54-0.95) | 0.83 (0.58-1.20) | 0.77 |
| >=65 years | 1.00 (Ref) | 0.77 (0.61-0.96) | 0.81 (0.59-1.09) | 1.39 (0.95-2.04) |  |
| **Green Tea** | | | | | |
| Overall | 1.00 (Ref) | 0.90 (0.77-1.06) | 0.73 (0.57-0.93) | 0.70 (0.54-0.90) | - |
| <65years | 1.00 (Ref) | 0.81 (0.64-1.01) | 0.70 (0.50-0.98) | 0.70 (0.50-0.98) | 0.65 |
| >=65 years | 1.00 (Ref) | 0.97 (0.74-1.27) | 0.79 (0.53-1.18) | 0.60 (0.38-0.95) |  |
| **Other Tea** | | | | | |
| Overall | 1.00 (Ref) | 0.84 (0.74-0.94) | 0.90 (0.77-1.06) | 0.79 (0.64-0.98) | - |
| <65years | 1.00 (Ref) | 0.84 (0.71-1.00) | 0.83 (0.67-1.04) | 0.79 (0.60-1.05) | 0.47 |
| >=65 years | 1.00 (Ref) | 0.86 (0.71-1.04) | 0.99 (0.77-1.29) | 0.75 (0.52-1.08) |  |

Conditional logistic regression models; Adjusted for age, ethnicity, education, occupation, BMI, physical activity, alcohol, smoking, diet (tertile), apob_apoa, diabetes, hypertension, cardiac risk factors, global stress, other beverage intake (coffee, black tea, green tea, other tea, water, fruit drink and carbonated beverage, as appropriate), adding milk to tea or coffee and the interaction between tea and coffee (all tea/coffee, black tea/coffee, green tea/coffee or othertea/coffee, as appropriate)

**Table S8. Association between Tea/Coffee Consumption and All Stroke – Stratified by Sex**

|  | **None** | **1-2cups/day** | **3-4 cups/day** | **>4cups/day** | **p_int_** |
| --- | --- | --- | --- | --- | --- |
| **Coffee** | | | | | |
| Overall | 1.00 (Ref) | 1.01 (0.88-1.15) | 1.07 (0.88-1.30) | 1.37 (1.06-1.77) | - |
| Male | 1.00 (Ref) | 1.02 (0.86-1.21) | 0.99 (0.78-1.27) | 1.42 (1.03-1.95) | 0.96 |
| Female | 1.00 (Ref) | 1.00 (0.81-1.25) | 1.25 (0.90-1.75) | 1.22 (0.77-1.93) |  |
| **All Tea** | | | | | |
| Overall | 1.00 (Ref) | 0.82 (0.73-0.92) | 0.80 (0.70-0.91) | 0.81 (0.69-0.94) | - |
| Male | 1.00 (Ref) | 0.81 (0.70-0.93) | 0.86 (0.73-1.02) | 0.84 (0.69-1.02) | 0.09 |
| Female | 1.00 (Ref) | 0.86 (0.72-1.03) | 0.70 (0.56-0.88) | 0.75 (0.57-1.00) |  |
| **Black Tea** | | | | | |
| Overall | 1.00 (Ref) | 0.71 (0.62-0.82) | 0.71 (0.58-0.86) | 1.03 (0.80-1.32) | - |
| Male | 1.00 (Ref) | 0.68 (0.57-0.82) | 0.65 (0.51-0.83) | 1.04 (0.76-1.42) | 0.79 |
| Female | 1.00 (Ref) | 0.79 (0.63-1.00) | 0.85 (0.62-1.16) | 1.05 (0.69-1.59) |  |
| **Green Tea** | | | | | |
| Overall | 1.00 (Ref) | 0.90 (0.77-1.06) | 0.73 (0.57-0.93) | 0.70 (0.54-0.90) | - |
| Male | 1.00 (Ref) | 1.04 (0.85-1.29) | 0.87 (0.65-1.16) | 0.76 (0.56-1.01) | 0.16 |
| Female | 1.00 (Ref) | 0.73 (0.56-0.95) | 0.49 (0.31-0.78) | 0.59 (0.33-1.04) |  |
| **Other Tea** | | | | | |
| Overall | 1.00 (Ref) | 0.84 (0.74-0.94) | 0.90 (0.77-1.06) | 0.79 (0.64-0.98) | - |
| Male | 1.00 (Ref) | 0.82 (0.70-0.96) | 1.02 (0.84-1.24) | 0.84 (0.66-1.08) | 0.07 |
| Female | 1.00 (Ref) | 0.87 (0.72-1.06) | 0.71 (0.54-0.94) | 0.69 (0.46-1.04) |  |

Conditional logistic regression models; Adjusted for age, ethnicity, education, occupation, BMI, physical activity, alcohol, smoking, diet (tertile), apob_apoa, diabetes, hypertension, cardiac risk factors, global stress, other beverage intake (coffee, black tea, green tea, other tea, water, fruit drink and carbonated beverage, as appropriate), adding milk to tea or coffee and the interaction between tea and coffee (all tea/coffee, black tea/coffee, green tea/coffee or othertea/coffee, as appropriate)

**Table S9. Association between Tea/Coffee Consumption and All Stroke – Stratified by Smoking**

|  | **None** | **1-2cups/day** | **3-4 cups/day** | **>4cups/day** | **p_int_** |
| --- | --- | --- | --- | --- | --- |
| **Coffee** | | | | | |
| Overall | 1.00 (Ref) | 1.01 (0.88-1.15) | 1.07 (0.88-1.30) | 1.37 (1.06-1.77) | - |
| Never/Former Smoker | 1.00 (Ref) | 1.02 (0.89-1.44) | 1.13 (0.89-1.44) | 1.46 (1.03-2.06) | 0.16 |
| Current Smoker | 1.00 (Ref) | 0.79 (0.43-1.46) | 0.65 (0.30-1.43) | 0.72 (0.28-1.89) |  |
| **All Tea** | | | | | |
| Overall | 1.00 (Ref) | 0.82 (0.73-0.92) | 0.80 (0.70-0.91) | 0.81 (0.69-0.94) | - |
| Never/Former Smoker | 1.00 (Ref) | 0.81 (0.70-0.93) | 0.76 (0.64-0.90) | 0.90 (0.72-1.11) | 0.49 |
| Current Smoker | 1.00 (Ref) | 0.68 (0.48-0.97) | 0.91 (0.62-1.35) | 0.54 (0.34-0.85) |  |
| **Black Tea** | | | | | |
| Overall | 1.00 (Ref) | 0.71 (0.62-0.82) | 0.71 (0.58-0.86) | 1.03 (0.80-1.32) | - |
| Never/Former Smoker | 1.00 (Ref) | 0.79 (0.66-0.94) | 0.79 (0.62-1.00) | 1.24 (0.90-1.69) | 0.12 |
| Current Smoker | 1.00 (Ref) | 0.60 (0.35-1.02) | 0.34 (0.15-0.73) | 0.55 (0.23-1.31) |  |
| **Green Tea** | | | | | |
| Overall | 1.00 (Ref) | 0.90 (0.77-1.06) | 0.73 (0.57-0.93) | 0.70 (0.54-0.90) | - |
| Never/Former Smoker | 1.00 (Ref) | 0.78 (0.63-0.97) | 0.60 (0.43-0.86) | 0.64 (0.43-0.96) | 0.34 |
| Current Smoker | 1.00 (Ref) | 1.24 (0.78-1.97) | 0.72 (0.38-1.37) | 0.43 (0.23-0.81) |  |
| **Other Tea** | | | | | |
| Overall | 1.00 (Ref) | 0.84 (0.74-0.94) | 0.90 (0.77-1.06) | 0.79 (0.64-0.98) | - |
| Never/Former Smoker | 1.00 (Ref) | 0.86 (0.74-1.00) | 0.90 (0.73-1.12) | 0.98 (0.73-1.32) | 0.15 |
| Current Smoker | 1.00 (Ref) | 0.78 (0.53-1.16) | 1.21 (0.78-1.89) | 0.68 (0.38-1.22) |  |

Conditional logistic regression models; Adjusted for age, ethnicity, education, occupation, BMI, physical activity, alcohol, smoking, diet (tertile), apob_apoa, diabetes, hypertension, cardiac risk factors, global stress, other beverage intake (coffee, black tea, green tea, other tea, water, fruit drink and carbonated beverage, as appropriate), adding milk to tea or coffee and the interaction between tea and coffee (all tea/coffee, black tea/coffee, green tea/coffee or othertea/coffee, as appropriate)

**Table S10. Association between Tea/Coffee Consumption and All Stroke – Stratified by Alcohol Consumption**

|  | **None** | **1-2cups/day** | **3-4 cups/day** | **>4cups/day** | **p_int_** |
| --- | --- | --- | --- | --- | --- |
| **Coffee** | | | | | |
| Overall | 1.00 (Ref) | 1.01 (0.88-1.15) | 1.07 (0.88-1.30) | 1.37 (1.06-1.77) | - |
| Never/Former Drinker | 1.00 (Ref) | 1.03 (0.86-1.23) | 1.51 (1.13-2.01) | 1.89 (1.25-2.86) | <0.001 |
| Current Drinker | 1.00 (Ref) | 0.92 (0.64-1.31) | 0.67 (0.41-1.10) | 0.88 (0.48-1.59) |  |
| **All Tea** | | | | | |
| Overall | 1.00 (Ref) | 0.82 (0.73-0.92) | 0.80 (0.70-0.91) | 0.81 (0.69-0.94) | - |
| Never/Former Drinker | 1.00 (Ref) | 0.83 (0.72-0.96) | 0.78 (0.66-0.93) | 0.85 (0.69-1.05) | 0.21 |
| Current Drinker | 1.00 (Ref) | 1.00 (0.74-1.34) | 0.95 (0.66-1.36) | 1.31 (0.85-2.02) |  |
| **Black Tea** | | | | | |
| Overall | 1.00 (Ref) | 0.71 (0.62-0.82) | 0.71 (0.58-0.86) | 1.03 (0.80-1.32) | - |
| Never/Former Drinker | 1.00 (Ref) | 0.74 (0.62-0.89) | 0.77 (0.59-0.99) | 1.19 (0.85-1.67) | 0.17 |
| Current Drinker | 1.00 (Ref) | 0.83 (0.57-1.22) | 0.73 (0.44-1.22) | 1.60 (0.82-3.09) |  |
| **Green Tea** | | | | | |
| Overall | 1.00 (Ref) | 0.90 (0.77-1.06) | 0.73 (0.57-0.93) | 0.70 (0.54-0.90) | - |
| Never/Former Drinker | 1.00 (Ref) | 0.87 (0.70-1.07) | 0.59 (0.43-0.82) | 0.51 (0.36-0.72) | 0.04 |
| Current Drinker | 1.00 (Ref) | 0.75 (0.45-1.23) | 1.55 (0.79-3.04) | 1.82 (0.97-3.42) |  |
| **Other Tea** | | | | | |
| Overall | 1.00 (Ref) | 0.84 (0.74-0.94) | 0.90 (0.77-1.06) | 0.79 (0.64-0.98) | - |
| Never/Former Drinker | 1.00 (Ref) | 0.91 (0.78-1.06) | 0.97 (0.79-1.19) | 0.95 (0.71-1.28) | 0.17 |
| Current Drinker | 1.00 (Ref) | 0.73 (0.51-1.04) | 0.82 (0.52-1.29) | 0.88 (0.49-1.56) |  |

Conditional logistic regression models; Adjusted for age, ethnicity, education, occupation, BMI, physical activity, alcohol, smoking, diet (tertile), apob_apoa, diabetes, hypertension, cardiac risk factors, global stress, other beverage intake (coffee, black tea, green tea, other tea, water, fruit drink and carbonated beverage, as appropriate), adding milk to tea or coffee and the interaction between tea and coffee (all tea/coffee, black tea/coffee, green tea/coffee or othertea/coffee, as appropriate)

**Table S11. Association between Tea/Coffee Consumption and All Stroke – Stratified by Hypertension Status**

|  | **None** | **1-2cups/day** | **3-4 cups/day** | **>4cups/day** | **p_int_** |
| --- | --- | --- | --- | --- | --- |
| **Coffee** | | | | | |
| Overall | 1.00 (Ref) | 1.01 (0.88-1.15) | 1.07 (0.88-1.30) | 1.37 (1.06-1.77) | - |
| No Hypertension | 1.00 (Ref) | 0.81 (0.56-1.16) | 0.58 (0.33-1.01) | 0.92 (0.46-1.84) | 0.001 |
| Hypertension | 1.00 (Ref) | 1.02 (0.85-1.23) | 1.17 (0.88-1.54) | 1.14 (0.78-1.68) |  |
| **All Tea** | | | | | |
| Overall | 1.00 (Ref) | 0.82 (0.73-0.92) | 0.80 (0.70-0.91) | 0.81 (0.69-0.94) | - |
| No Hypertension | 1.00 (Ref) | 0.69 (0.51-0.93) | 0.96 (0.69-1.33) | 0.75 (0.49-1.14) | 0.03 |
| Hypertension | 1.00 (Ref) | 0.73 (0.60-0.90) | 0.68 (0.51-0.90) | 1.07 (0.73-1.56) |  |
| **Black Tea** | | | | | |
| Overall | 1.00 (Ref) | 0.71 (0.62-0.82) | 0.71 (0.58-0.86) | 1.03 (0.80-1.32) | - |
| No Hypertension | 1.00 (Ref) | 0.51 (0.35-0.75) | 0.80 (0.48-1.34) | 0.61 (0.29-1.28) | 0.003 |
| Hypertension | 1.00 (Ref) | 0.67 (0.55-0.80) | 0.58 (0.46-0.75) | 0.88 (0.63-1.22) |  |
| **Green Tea** | | | | | |
| Overall | 1.00 (Ref) | 0.90 (0.77-1.06) | 0.73 (0.57-0.93) | 0.70 (0.54-0.90) | - |
| No Hypertension | 1.00 (Ref) | 1.16 (0.74-1.83) | 0.69 (0.39-1.23) | 0.99 (0.51-1.92) | 0.74 |
| Hypertension | 1.00 (Ref) | 0.80 (0.63-1.02) | 0.65 (0.44-0.97) | 0.71 (0.46-1.09) |  |
| **Other Tea** | | | | | |
| Overall | 1.00 (Ref) | 0.84 (0.74-0.94) | 0.90 (0.77-1.06) | 0.79 (0.64-0.98) | - |
| No Hypertension | 1.00 (Ref) | 0.74 (0.53-1.04) | 1.22 (0.82-1.83) | 1.04 (0.60-1.81) | 0.93 |
| Hypertension | 1.00 (Ref) | 0.74 (0.62-0.88) | 0.68 (0.53-0.87) | 0.66 (0.48-0.91) |  |

Conditional logistic regression models; Adjusted for age, ethnicity, education, occupation, BMI, physical activity, alcohol, smoking, diet (tertile), apob_apoa, diabetes, hypertension, cardiac risk factors, global stress, other beverage intake (coffee, black tea, green tea, other tea, water, fruit drink and carbonated beverage, as appropriate), adding milk to tea or coffee and the interaction between tea and coffee (all tea/coffee, black tea/coffee, green tea/coffee or othertea/coffee, as appropriate)

**Table S12. Association between Tea/Coffee Consumption and All Stroke – Stratified by Addition of Milk to Tea/Coffee**

|  | **None** | **1-2cups/day** | **3-4 cups/day** | **>4cups/day** | **p_int_** |
| --- | --- | --- | --- | --- | --- |
| **Coffee** | | | | | |
| Overall | 1.00 (Ref) | 1.01 (0.88-1.15) | 1.07 (0.88-1.30) | 1.37 (1.06-1.77) | - |
| No Added Milk | 1.00 (Ref) | 1.18 (0.97-1.45) | 1.35 (1.03-1.79) | 1.51 (1.05-2.16) | 0.07 |
| Added Milk | 1.00 (Ref) | 0.93 (0.70-1.24) | 0.85 (0.53-1.36) | 1.63 (0.86-3.09) |  |
| **All Tea** | | | | | |
| Overall | 1.00 (Ref) | 0.82 (0.73-0.92) | 0.80 (0.70-0.91) | 0.81 (0.69-0.94) | - |
| No Added Milk | 1.00 (Ref) | 0.86 (0.75-0.99) | 0.66 (0.56-0.79) | 0.66 (0.54-0.81) | <0.01 |
| Added Milk | 1.00 (Ref) | 1.14 (0.77-1.69) | 1.31 (0.86-2.00) | 1.89 (1.17-3.07) |  |
| **Black Tea** | | | | | |
| Overall | 1.00 (Ref) | 0.71 (0.62-0.82) | 0.71 (0.58-0.86) | 1.03 (0.80-1.32) | - |
| No Added Milk | 1.00 (Ref) | 0.66 (0.53-0.81) | 0.46 (0.34-0.63) | 0.85 (0.59-1.22) | 0.03 |
| Added Milk | 1.00 (Ref) | 0.81 (0.61-1.09) | 1.08 (0.74-1.59) | 1.70 (1.03-2.82) |  |
| **Green Tea** | | | | | |
| Overall | 1.00 (Ref) | 0.90 (0.77-1.06) | 0.73 (0.57-0.93) | 0.70 (0.54-0.90) | - |
| No Added Milk | 1.00 (Ref) | 0.87 (0.71-1.05) | 0.69 (0.52-0.90) | 0.71 (0.54-0.94) | 0.79 |
| Added Milk | 1.00 (Ref) | 0.67 (0.38-1.19) | 0.98 (0.36-2.65) | 1.12 (0.23-5.41) |  |
| **Other Tea** | | | | | |
| Overall | 1.00 (Ref) | 0.84 (0.74-0.94) | 0.90 (0.77-1.06) | 0.79 (0.64-0.98) | - |
| No Added Milk | 1.00 (Ref) | 0.91 (0.78-1.08) | 0.80 (0.63-1.00) | 0.55 (0.40-0.75) | <0.01 |
| Added Milk | 1.00 (Ref) | 0.83 (0.60-1.15) | 0.97 (0.68-1.38) | 1.25 (0.82-1.89) |  |

Conditional logistic regression models; Adjusted for age, ethnicity, education, occupation, BMI, physical activity, alcohol, smoking, diet (tertile), apob_apoa, diabetes, hypertension, cardiac risk factors, global stress, other beverage intake (coffee, black tea, green tea, other tea, water, fruit drink and carbonated beverage, as appropriate), adding milk to tea or coffee and the interaction between tea and coffee (all tea/coffee, black tea/coffee, green tea/coffee or othertea/coffee, as appropriate)

**Table S13. Association between Tea/Coffee Consumption and All Stroke Excluding Proxy Assistance**

|  | **None** | **1-2cups/day** | **3-4 cups/day** | **>4cups/day** |
| --- | --- | --- | --- | --- |
| **Coffee** | | | | |
| All Participants | 1.00 (Ref) | 0.96 (0.86-1.08) | 1.00 (0.85-1.19) | 1.27 (1.01-1.60) |
| Excluding Proxy Assistance | 1.00 (Ref) | 0.89 (0.73-1.06) | 0.81 (0.63-1.05) | 1.22 (0.88-1.69) |
| **All Tea** | | | | |
| All Participants | 1.00 (Ref) | 0.79 (0.71-0.87) | 0.76 (0.67-0.85) | 0.76 (0.66-0.88) |
| Excluding Proxy Assistance | 1.00 (Ref) | 0.80 (0.68-0.93) | 0.69 (0.55-0.81) | 0.64 (0.51-0.81) |
| **Black Tea** | | | | |
| All Participants | 1.00 (Ref) | 0.69 (0.60-0.78) | 0.68 (0.57-0.80) | 0.98 (0.78-1.22) |
| Excluding Proxy Assistance | 1.00 (Ref) | 0.71 (0.62-0.82) | 0.71 (0.58-0.86) | 1.03 (0.80-1.32) |
| **Green Tea** | | | | |
| All Participants | 1.00 (Ref) | 0.89 (0.76-1.03) | 0.72 (0.57-0.91) | 0.69 (0.54-0.88) |
| Excluding Proxy Assistance | 1.00 (Ref) | 0.92 (0.74-1.14) | 0.62 (0.45-0.85) | 0.62 (0.44-0.87) |
| **Other Tea** | | | | |
| All Participants | 1.00 (Ref) | 0.80 (0.72-0.89) | 0.86 (0.74-0.99) | 0.75 (0.61-0.91) |
| Excluding Proxy Assistance | 1.00 (Ref) | 0.78 (0.65-0.93) | 0.78 (0.61-1.01) | 0.58 (0.42-0.81) |

Conditional logistic regression models; Adjusted for age, ethnicity, education, occupation, BMI, physical activity, alcohol, smoking, diet (tertile), apob_apoa, diabetes, hypertension, cardiac risk factors, global stress, other beverage intake (coffee, black tea, green tea, other tea, water, fruit drink and carbonated beverage, as appropriate), adding milk to tea or coffee and the interaction between tea and coffee (all tea/coffee, black tea/coffee, green tea/coffee or othertea/coffee, as appropriate)
